# 

**Published:** 1889-12-21

**Authors:** 


PRICE ONE PENNY.

. 169, Vol. VII.—December 21st, 1889.

[Registered at the General Post Office as a Newspaper for transmission in the United Kingdom and Abroad.

WITH EXTRA NURSING SUPPLEMENT.
Per Annum, 6s. 6d., post free.
Monthly Parts, 6d.; post free 7½d.
Ditto per Annum, 7s. 6d., post free.

